# Copper depletion inhibits CoCl_2_-induced aggressive phenotype of MCF-7 cells via downregulation of HIF-1 and inhibition of Snail/Twist-mediated epithelial-mesenchymal transition

**DOI:** 10.1038/srep12410

**Published:** 2015-07-15

**Authors:** Shun Li, Jing Zhang, Hong Yang, Chunhui Wu, Xitong Dang, Yiyao Liu

**Affiliations:** 1 Department of Biophysics, School of Life Science and Technology; 2 Center for Information in Biomedicine, University of Electronic Science and Technology of China, Chengdu 610054, Sichuan, P.R. China; 3 Division of Trauma, Surgical Critical Care and Burns, University of California San Diego, CA 92103, USA

## Abstract

Copper, a strictly regulated trace element, is essential for many physiological processes including angiogenesis. Dysregulated angiogenesis has been associated with increased copper in tumors, and thus copper chelators have been used to inhibit tumor angiogenesis. However, it remains unclear whether copper has any effect on epithelial-mesenchymal transition (EMT). Using CoCl_2_-induced EMT of human breast carcinoma MCF-7 cells, we found that TEPA, a copper chelator, inhibited EMT-like cell morphology and cytoskeleton arrangement triggered by CoCl_2_; decreased the expression of vimentin and fibronectin, markers typical of EMT; inhibited HIF-1 activation and HIF1-α accumulation in nuclear; and down-regulated the expression of hypoxia-associated transcription factors, Snail and Twist1. Moreover, knockdown copper transport protein, *Ctr1*, also inhibited CoCl_2_-induced EMT and reversed the mesenchymal phenotype. In EMT6 xenograft mouse models, TEPA administration inhibited the tumor growth and increased mice survival. Immunohistochemical analysis of the xenograft further demonstrated that TEPA administration significantly inhibited tumor angiogenesis, down-regulated hypoxia-induced transcription factors, Snail and Twist1, leading to decreased transactivation of EMT-associated marker genes, vimentin and fibronectin. These results indicate that TEPA inhibits CoCl_2_-induced EMT most likely via HIF1-α-Snail/Twist signaling pathway, and copper depletion may be exploited as a therapeutic for breast cancer.

Copper, an essential mineral, regulates many physiological functions including blood pressure, heart rate, neuronal function, energy metabolism, and the absorption of other nutrients. However, aberrantly increased copper has been shown to promote angiogenesis^1^,^2^, which agrees with the discoveries that copper regulated directly or indirectly numerous angiogenesis-related factors^3^. Accumulating scientific evidence shows that copper plays a critical role in the progression of cancers of brain, breast, colon, lung and prostate^4^,^5^,^6^,^7^,^8^. A national cohort of United States adults concluded that patients with a higher level of serum copper had a significantly increased risk of dying from cancer^9^.

Growth of tumors generally depends on the development of the tumor’s own vasculature, thus anti-angiogenic reagents that inhibit genesis of new blood vessels have become one of the promising treatment strategies for cancer^10^,^11^,^12^,^13^. As a physiological angiogenic factor that aberrantly increased in tumors, copper would be an ideal target for inhibiting the progression of tumors and thus increasing survival time^14^. Indeed, copper chelators that deplete free copper suppressed tumor growth in animal models and several clinical trials of different tumors^15^. Although the mechanisms of tumor-inhibiting effect of copper depletion are attributed mainly to its effect on angiogenesis, increasing evidence shows that the treatment effect of copper depletion may come from other mechanisms. In a phase II clinical trial, treatment of patients with high risk recurrence of breast cancer suggested that copper chelator appeared to maintain endothelial progenitor cells (EPCs) below baseline to promote tumor dormancy and ultimately prevent relapse^16^. Reducing systemic copper level decreased the activity of the copper-binding mitochondrial enzyme cytochrome c oxidase and reduced ATP levels, which led to decreased oxidative phosphorylation in rapidly proliferating cancer cells in pancreatic cancer^17^. Decreasing the levels of copper transporter-1 (*Ctr1*) or mutation of mitogen-activated protein kinase kinase-1 (MEK-1) that disrupt copper binding, suppressed the mitogen-activated protein kinase (MAPK) pathway in both mouse and human cell settings^18^,^19^.

Epithelial-mesenchymal transition (EMT) is characterized by epithelial cells losing their cell polarity and cell-cell adhesion, and gaining migratory and invasive properties to transform to mesenchymal stem cells. EMT was originally used to describe cell remodeling during heart morphogenesis, later it is found to be involved in the processes of mesoderm and neural crest formation. During tumor progression, EMT is reactivated by some undefined molecular cues. Hypoxia, a common characteristic of solid tumors, has been reported to reactivate EMT through hypoxia-induced factor-1 alpha (HIF-1α) in several tumor models^20^,^21^,^22^,^23^. HIF-1α regulates directly or indirectly EMT regulators that include including Snail, Twist1, Slug, ZEB1, ZEB2, and E12/E47, and other transcription factors^24^,^25^,^26^,^27^, and these transcription factors then transactivate EMT-related genes such as E-cadherin, Vimentin, and N-cadherin to mediate EMT^28^,^29^,^30^,^31^. Other than activation of HIF-1α-Snail/Twist signaling pathway, hypoxia also has been shown to regulate TGFβ signaling pathway, chromatin modification enzymes, and other molecules that promote tumor progression in an EMT-dependent or –independent manners^20^,^21^,^22^,^23^.

Although it is well known for its potent redox activity and its critical role in cellular respiration and antioxidant activity, copper as a regulator of cell signaling has just begun to be realized. Reports have shown that copper level was aberrantly up-regulated in many tumors, and depletion of which resulted in suppression of many cell signaling pathways including Hypoxia-HIF-1α, NF-κB, ERK, p38, and JNK, among others, leading to inhibition of tumor growth and reduced metastasis^24^,^25^,^26^. In our previous study, we found copper was involved in cell cytoskeleton rearrangement and migration^32^, which was closely associated with EMT and migration of cancer cells. High levels of copper can stabilize HIF-1α and thus increase its activity, which in turn up-regulates its downstream EMT regulators, leading to transactivation of EMT marker genes including N- and E-cadherin and vimentin^25^,^26^. It was reported that hypoxia can also induce EMT of breast cancer cells through regulation of EMT regulators^33^. It is thus important to clarify whether copper chelator could directly affect hypoxia-induced EMT in cancer cells.

We hypothesized that copper chelation may regulate the remodeling of cytoskeleton and inhibit EMT related transcription factors, consequently leading to down-regulation of genes promoting EMT of human breast cancer cells. To this end, CoCl_2_, a mimic of hypoxia, was used to induce EMT. Copper chelator, tetraethylenepentamine (TEPA) was used to deplete copper, and the effect of TEPA on CoCl_2_-induced EMT was studied both *in vivo* and *in vitro*. We report that TEPA treatment reversed the EMT phenotype; down-regulated the expression of many EMT-related genes including vimentin, fibronectin and transcription factors, Snail and Twist-1; inhibited cell adhesion, migration, and invasion; halted tumor growth and progression; and significantly enhanced the survival of mouse xenografted with EMT6 breast cancer cells. Our study provides new mechanistic insight that TEPA inhibits tumor progression through reversal of EMT and down-regulation of HIF1-α-Snail/Twist signal pathway.

## Materials and Methods

### Animals

BALB/c mice were purchased from the Chengdu DaShuo laboratory animals Co., Ltd. (License number SCXK2014-189). The mice were kept in cages under standard laboratory conditions, fed with standard chow diet, and allowed free access to water. All experimental procedures were approved by the Ethic Committee of the University of Electronic Science and Technology of China in accordance with the Principles of Laboratory Animal Care formulated by the National Society for Medical Research.

### Cell culture and treatment

MCF-7 and EMT6 cells, obtained from the American Type Culture Collection (Manassas, VA), were maintained in RPMI 1640 (Gibco, New York, NY, USA) supplemented with 10% FBS (Gibco, Mulgrave, VIC, Australia) and 1% antibiotic-antimycotic (Gibco, New York, NY) in a standard culture incubator with humidified air containing 5% CO_2_ at 37 °C. Cells were seeded at 1.5 × 10^6^ per well in 6-well plates or 3.5 mm dishes the day before experiments. The next day, 200 μM CoCl_2_ (Kelong, Chengdu, China) and/or 50 μM tetraethylenepentamine (TEPA) (Sigma, St Louis, MO) was added to the cultures, which were continued to incubate for different time periods as indicated.

### Cell polarity assay

Cells were treated with CoCl_2_ and/or TEPA at the concentrations indicated for 24 h, and images were acquired. Cell polarity was analyzed using Image-pro plus 6.0. Both the lengths of the minor axis (X axis) and major axis (Y axis) were measured, and the ratio of Y to X was used to evaluate the cell polarity. In each experiment, more than 260 cells from each group were measured.

### Phalloidin staining

After 24 h culture, cells were fixed with 4% formalin for 20 min at room temperature, washed three times with phosphate buffered saline (PBS), and stained with fluorescent phalloidin conjugate solution (5 μg/ml) (Sigma, Carlsbad, CA, USA) in PBS for 40 min at 37 °C. The unbound phalloidin conjugate was then washed away with PBS for three times. Images were acquired under confocal laser scanning microscope (Nikon A1, Tokyo, Japan).

### Real time-PCR

After the specified treatments, cells were harvested and total RNA was extracted using RNAiso Plus kit (Takara, Shiga, Japan). Total RNA was reverse transcribed using PrimeScript^TM^ RT reagent kit (Takara) following the recommended conditions: 37 °C for 15 min, 85 °C for 5 s, and hold at 4 °C. cDNA corresponding to 100 ng of RNA was used for real-time PCR using a SYBR^®^ Premix Ex Taq™ II kit (Takara) on CFX96 Touch™ Real-Time PCR Detection System (Bio-Rad, Benicia, CA, USA). The real-time PCR conditions were 95 °C for 30 s, followed by 40 cycles of 95 °C for 5 s and 60 °C for 20 s. Primers for human β-actin, vimentin, vascular endothelial growth factor A (VEGFA), fibronectin, Snail, and Twist1 were listed in Table S1 (see supporting information). Efficiency of all primer pairs were >95%. Gene expression normalized to that of β-actin was calculated using 2-ΔΔCt method. Data were presented as fold-changes.

### Protein extraction and Western blotting analysis

Cells were rinsed with PBS and lysed with RNAiso Plus (Takara, Shiga, Japan). Protein concentrations were determined using DC^TM^ Protein Assay (Bio-Rad, CA). Ten μg of lysates were resolved on a 10% SDS-PAGE and transferred to PVDF membranes. The membranes were blocked with Tris-buffered saline (TBS) containing 5% non-fat dry milk and 0.05% Tween-20 at room temperature for 1 h, incubated with primary antibodies for human E-cadherin (Santa Cruz Biotech, CA), vimentin (Santa Cruz Biotech), fibronectin (Santa Cruz Biotech), Snail (Santa Cruz Biotech), Twist1 (Santa Cruz Biotech), HIF1-α (Abcam, Cambridge, UK), factor-inhibiting HIF-1 (FIH-1) (Abcam) and β-actin (Santa Cruz Biotech), respectively at 4 °C for overnight. The membranes were then incubated with corresponding secondary antibodies at room temperature for 1 h. The immunoreactive bands were developed using Immobilon Western Chemiluminescent HRP Substrate (Millipore, Billerica, MA, USA), and densitometric analysis was carried out using Quantity One software (Bio-Rad).

### Immunofluorescence

MCF-7 cells grown on coverslips were fixed with 4% formaldehyde for 15 min at room temperature, permeabilized with 0.4% Triton X-100 for 15 min, and blocked with 1% bovine serum albumin (BSA) for 1 h. The cells were incubated with primary antibodies at 1:50 dilution at 4 °C for overnight, washed with PBS, and incubated with secondary antibody for 1 h at 37 °C. Slides were washed with PBS and counter-stained with DAPI. Fluorescent images were acquired with Nikon eclipse E600 microscope, and exported for quantification analysis. The fluorescence density was measured by Image-Pro Plus 6.0.

### Wound healing assay

MCF-7 cells were seeded at 1.5 × 10^6^ cells/well in 6-well plates. After cells were confluent, wound was created by scratching using a sterile pipette tip. The medium was aspirated off and displaced cells were washed with PBS. The media containing either CoCl_2_ and/or TEPA was refreshed, and images were acquired immediately (0 h) and at 24 h (24 h).

### Cell adhesion assay

After treatments with CoCl_2_ and/or TEPA for 24 h, cells were trypsinized, washed and re-suspended in 10% RPMI 1640. 1 × 10^4^ cells were added into each well and incubated for 6 h. The non-adherent cells were washed off with PBS and images were acquired using an inverted fluorescence microscope (Nikon TE-2000, Tokyo, Japan).

### Cell invasion assay

MCF-7 cells were trypsinized and re-suspended in 10% RPMI 1640. 5 × 10^3^ cells were seeded into the Matrigel-coated transwell chambers with 8-μm pore size (BD Biosciences, CA). The transwell chambers were then placed into wells of a 24-well plate that contained CoCl_2_ and/or TEPA supplemented media. After 24 h incubation, the cells on upper surface of the transwell were wiped out with cotton swabs and the invaded cells on the other side of the transwell were fixed with 4% formaldehyde and stained with DAPI. The transmigrated cells were counted in three random microscope fields.

### Gene silencing of Ctr1

siRNA targeting human *Ctr1* and scrambled control siRNA were designed and synthesized by RiboBio (RiboBio, Guangzhou, China). The sequences for siRNA targeting *Ctr1* were presented in Table S1. MCF-7 Cells were transfected with 10 nM annealed *Ctr1*-siRNA in medium free of FBS and antibiotics using Lipofectamine2000 (Invitrogen, CA, USA) according to the manufacturer’s instruction. Medium was refreshed 5 h after transfection. The cells were continued to incubate for a total of 24 h for the downstream experiments.

### HIF-1 activation assay

After 24-h culture, MCF-7 cells were washed with PBS. HIF-1 activation was evaluated by the HIF-1 activation assay kit (Active Motif, CA) following the manufacturer’s instruction. Absorbance was measured at 450 nm on Spectra Max190 (Molecular Devices, Sunnyvale, CA).

### *In vivo* anti-tumor studies

Female BALB/c mice with an average weight of 20–25 g were subcutaneously inoculated with 1 × 10^6^ EMT6 cells. The mice were then randomized into two groups (12 each). For TEPA treatment, 250 mg of TEPA was added in 1 L of drinking water (the daily intake would be 1 mg/mice based on the daily water consumption of 4 ml/mice). Water was prepared freshly every week. Tumor size was measured externally daily using calipers during the experimental period. Tumor volume was calculated at 1, 2, 3, and 4 weeks using the following equation: volume = ab^2^/2, where “a” is the length of the major axis and “b” is the length of the minor axis. After the last measurement, the animals were sacrificed and tumors will be processed for histology and immunohistochemistry (HIC).

### Histological and immunohistochemical assays

The tumor tissues were fixed in 4% formalin-PBS solution, embedded in paraffin, and cut into 5-μm thick sections. The sections were stained with hematoxylin and eosin (HE). For immunohistochemistry, the sections were stained with antibodies against Snail, Twist1, E-cadherin, and fibronectin respectively. All quantification was carried out by an observer blinded to the study under Nikon Eclipse TE600 microscope.

### Statistical analysis

All the data were obtained from three independent experiments, and expressed as means ± SEM. One-way ANOVA was used to compare data among experiment groups and the difference between groups was estimated by the least significant difference (LSD) test. *p* < 0.05 was considered statistically significant.

## Results

### Effect of CoCl_2_-induced hypoxia on EMT of MCF-7 cell line

**Figure 1:**
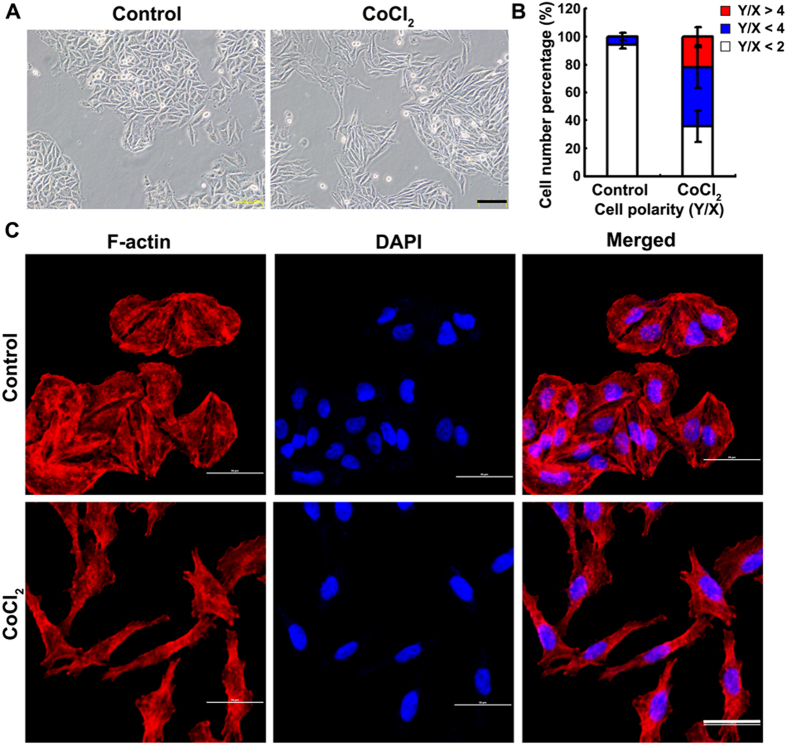
CoCl_2_-induced aggressive morphologic changes of MCF-7. (**A**) 200 μM CoCl_2_ increased spindle-shaped cells, but not control cells. Scale bars= 100 μm. (**B**) The morphology of MCF-7 cells was evaluated using polarity assay by comparing the ratio of major axis/minor axis. (**C**) Cell morphology was further confirmed by cell cytoskeletal staining. Cells were stained with TRITC-phalloidin which is specific for cellular F-actin. Scale bar = 50 μm.

**Figure 2:**
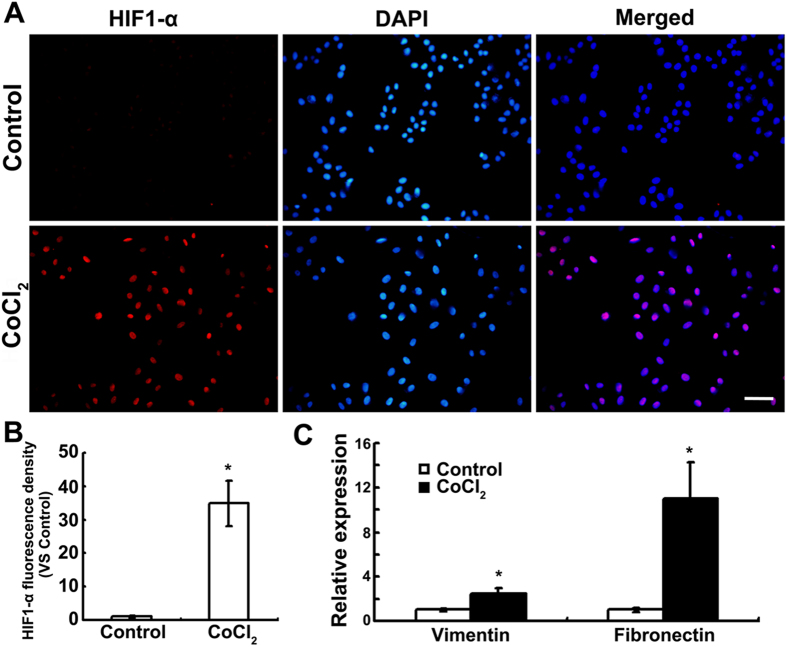
Expression of HIF1-α and EMT marker genes in MCF-7 cells under CoCl_2_ treatment. (**A**) Expression of HIF1-α after 24 h incubation with 200 μM CoCl_2_ or not were evaluated by indirect immunofluorescence staining. Scale bars = 100 μm. (**B**) The fluorescence density of HIF1-α was measured in each groups. Data was shown as mean ± SD of three independent experiments. (**C**) Expression of vimentin and fibronectin by real time-PCR after 24-h culture. Each experiment consisted of triplicate samples. ^*^p <0.05 was considered statistically significant.

Human breast cancer cell line MCF-7 exhibits a cobble-stone-like morphology typical of epithelial cells in normoxia. The cells lost their cell-cell contacts and the typical morphology of epithelial cells, and transformed into more elongated, spindle-like mesenchymal appearance with 200 μM CoCl_2_ supplement (Fig. 1A), which can be observed better with TRITC-phalloidin staining (Fig. 1C). Cell polarity, ratio of major axis to minor axis, was significantly increased by CoCl_2_ supplement, with 60% cells showing polarity >2 and 22% cells showing polarity >4 (Fig. 1B), compared to that <2 under normoxia. Hypoxia induced by CoCl_2_ was confirmed by immunofluorescence, showing increased nuclear localization of HIF1-α (Fig. 2A) and up-regulated HIF1-α expression (Fig. 2B). In agreement with the fibroblast-like mesenchymal transition triggered by CoCl_2_, the mRNA expression of mesenchymal markers, vimentin and fibronectin, was significantly up-regulated (Fig. 2C). These data indicate that CoCl_2_-induced hypoxia could trigger EMT of MCF-7 cells.

### TEPA inhibits CoCl_2_-induced EMT of MCF-7 cells

**Figure 3:**
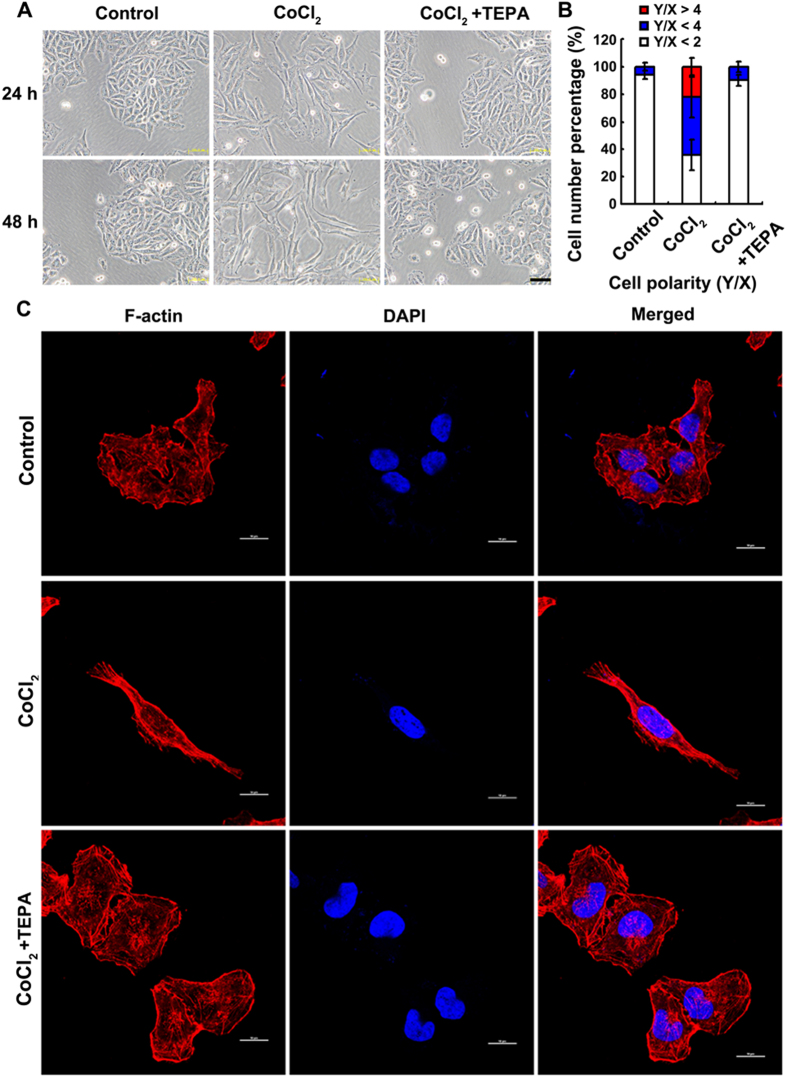
TEPA inhibits the CoCl_2_-induced morphologic changes of MCF-7 cells. (**A**) Few spindle-shaped cells were observed in cells treated with 50 μM TEPA supplement. Scale bars = 100 μm. (**B**) The morphology of MCF-7 cells was evaluated using polarity assay by comparing the ratio of major axis/minor axis. (**C**) Cell morphology was further evaluated by cell cytoskeletal staining. Cells were stained with TRITC-phalloidin which is specific for cellular F-actin. Scale bar= 50 μm.

**Figure 4:**
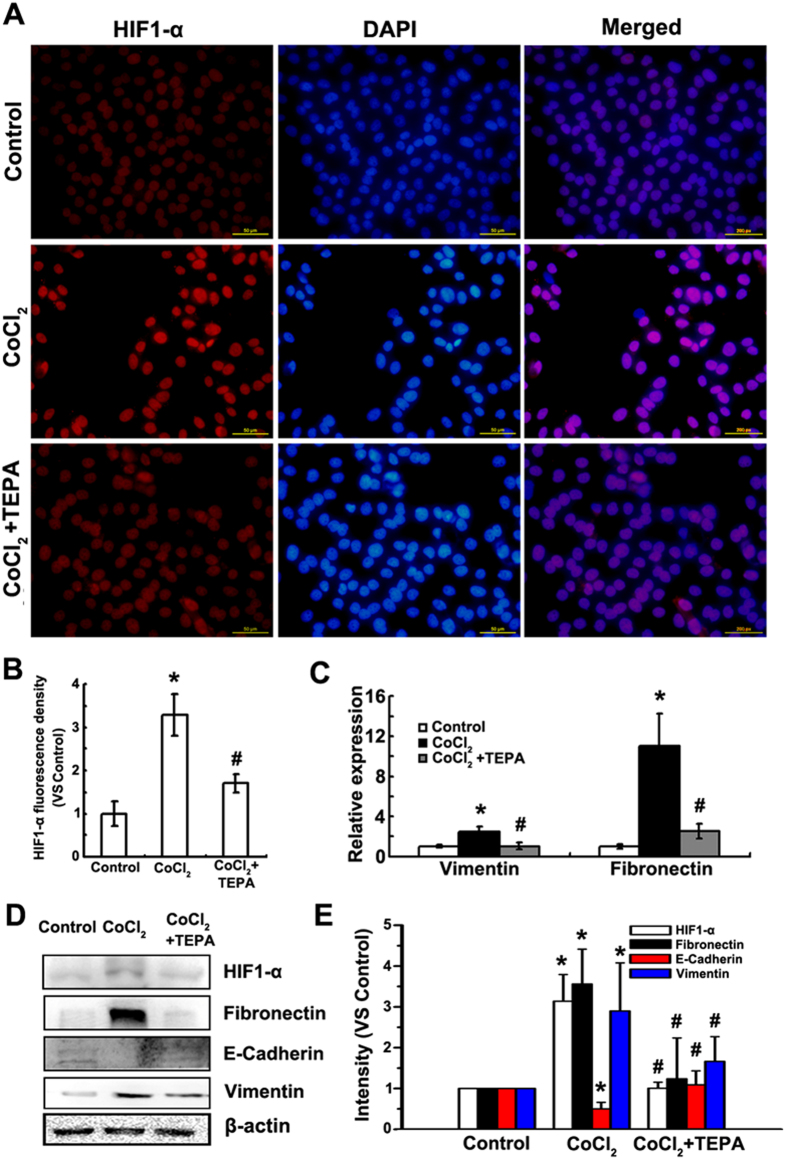
TEPA inhibits CoCl_2_-induced expression of HIF1-α and EMT marker genes in MCF-7 cells. (**A**) Expression of HIF1-α after 24 h incubation under control, 200 μM CoCl_2_ or treatment with 50 μM TEPA in MCF-7 cells by immunofluorescence. Scale bars = 100 μm. (**B**) The fluorescence density of HIF1-α was measured in each groups. Data was shown as mean ± SD of three independent experiments. (**C**) Expression of vimentin and fibronectin by real time-PCR after 24 h culture. Each experiment consisted of triplicate. (**D**) Expression of HIF1-α, fibronectin, E-cadherin and vimentin was detected by Western blotting. (**E**) The histograms showed the quantitative results of the target proteins expression, which were normalized with β-actin and expressed as fold change of control. Data was shown as mean ± SD of three independent experiments. *p* <0.05 was considered statistically significant (^*^compared to the control group and ^#^compared to the CoCl_2_ group).

**Figure 5:**
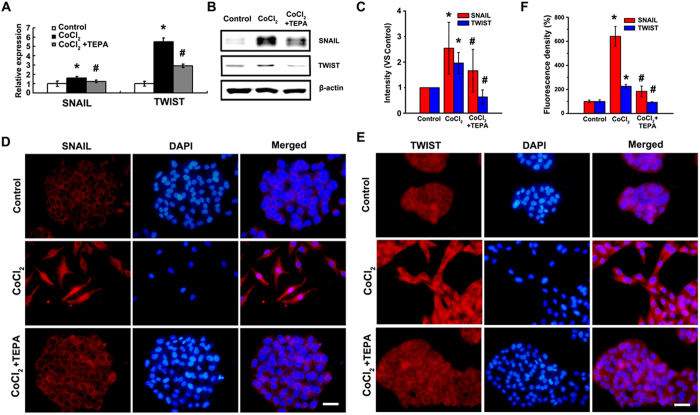
TEPA affects the expression of typical EMT marker genes. (**A**) Expression of E-cadherin in MCF-7 maintained for 24 h under control, 200 μM CoCl_2_ conditions or treatment with 50 μM TEPA. Scale bars = 50 μm. (**B**) Expression of vimentin in MCF-7 maintained for 24 h under normoxic condition, hypoxia condition or treatment with TEPA. Scale bars = 50 μm.

To assess effects of copper chelator on CoCl_2_-induced EMT of MCF-7 cells, TEPA, a specific copper chelator, was used to deplete the copper in culture medium. After 24-h treatment with TEPA (50 μM), few elongated and spindle-like mesenchyme-like cells were observed in either control or TEPA-treated groups (Fig. 3A). In contrast, cells under hypoxia-induced by CoCl_2_ (200 μM) exhibited epithelial cobblesone-like morphology (Fig. 3A). Similar results were obtained by quantitative evaluation of cell polarity. Copper depletion with TEPA showed 10% cells with polarity value above 2, which was similar to 6% cells with polarity value above 2 in control group (Fig. 3B). Cytoskeleton rearrangement and the formation of stress fibers were observed under oil objective (Fig. 3C). After 24 h treatment with CoCl_2_, cells exhibited typical fibroblast-like phenotype with actin filaments bundled into thick contractile stress fibers at the ventral cell surface, whereas cells treated with both CoCl_2_ and TEPA showed cortical thin bundles of actin filaments that were typical in control group. Furthermore, compared to cells treated with CoCl_2_, TEPA treatment reduced both nuclear (Fig. 4A,B) and total (Fig. 4D,E) accumulation of HIF1-α; significantly increased the expression of E-cadherin, an epithelial marker (Fig. 4D,E); and significantly decreased the expression of vimentin and fibronectin, markers of mesenchymal cells, at both mRNA and protein levels (Fig. 4C–E) as evaluated by both real-time PCR and Western blotting, respectively. Moreover, immunofluorescence data (Fig. 5) and quantitative analysis (Fig. S1) also demonstrated TEPA treatment increased the expression of E-cadherin and down-regulated expression of vimentin. Taken together, these results suggest that TEPA inhibited CoCl_2_-induced EMT of MCF-7 cells.

### TEPA inhibits CoCl_2_-induced migration, adhesion and invasion of MCF-7 cells

**Figure 6:**
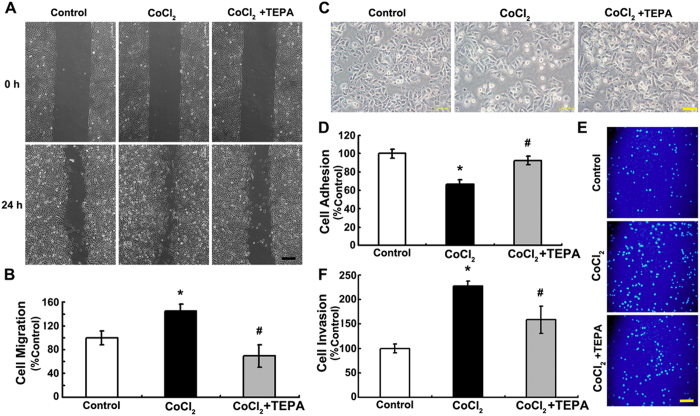
TEPA inhibits migration, adhesion and invasion of MCF-7 cells. (**A**) MCF-7 cells were incubated under control, 200 μM CoCl_2_ or CoCl_2_ with 50 μM TEPA treatment for 24 h, followed by a scratching assay. The relative wound closure was observed under microscope and photographed. Scale bars = 200 μm. (**B**) Quantification of cell motility by measuring the wound width. (**C**) After exposed to CoCl_2_ or CoCl_2_ with TEPA treatment for 24 h, MCF-7 cells were trypsinized and re-plated. Microscopic view of cells was taken after 6-h culture. Scale bars = 50 μm. (**D**) Quantification of cell adhesion. (**E**) Transwell assay of MCF-7 cells. Shown were representative fields of invasive and migratory cells on the membrane. Scale bars = 100 μm. (**F**) Quantification of cell invasion. All the experiments were performed using triplicate wells for each treatment condition and repeated three times. *p* < 0.05 was considered statistically significant (^*^compared to the control group and ^#^compared to the CoCl_2_ group).

To determine whether TEPA treatment had any effect on the behaviors of cells, wound healing, cell adhesion, and matrigel-coated transwell invasion assays were performed. The results showed that cells with 200 μM CoCl_2_ supplement migrated faster than the control or treated with 50 μM TEPA (Fig. 6A,B), which suggests that CoCl_2_ treatment promotes cell motility whereas TEPA inhibits CoCl_2_-induced cell motility. TEPA treatment also significantly inhibited cell invasiveness and adhesion induced by CoCl_2_ (Fig. 6C–F). These data demonstrate that copper depletion by TEPA inhibited the malignant phenotype of MCF-7 cells by reversing EMT and suppressing the motility, cell-cell adhesion and invasive ability.

### Ctr1 silencing inhibits CoCl_2_-induced cytoskeleton rearrangement and EMT marker genes expression

**Figure 7:**
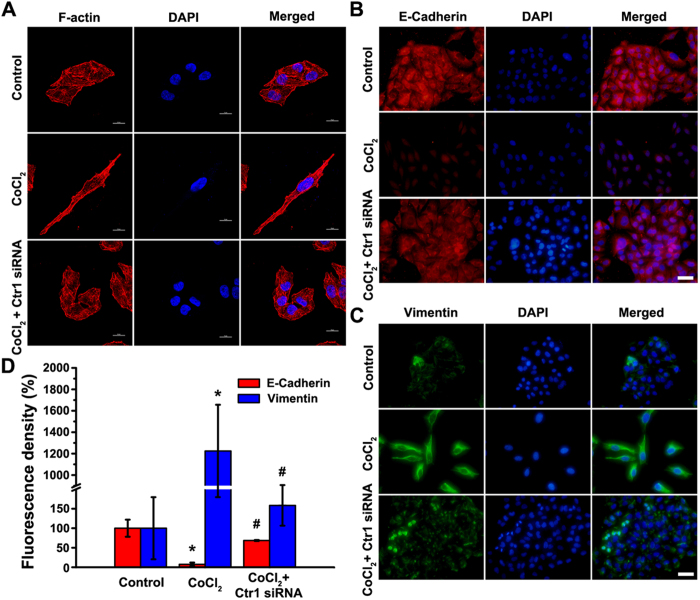
*Ctr1* siRNA inhibits CoCl_2_-induced cytoskeleton remodeling and the expression of EMT marker genes. (**A**) Representative images of fluorescently stained actin of MCF-7 cells under control, 200 μM CoCl_2_ or CoCl_2_ with *Ctr1* siRNA treatment. Scale bars = 10 μm. (**B**) Expression of E-cadherin in MCF-7 cells maintained for 24 h under control, 200 μM CoCl_2_ or CoCl_2_ with *Ctr1* siRNA treatment by indirect immunofluorescence staining. Scale bars = 50 μm. (**C**) Expression of vimentin in MCF-7 cells maintained for 24 h under control, 200 μM CoCl_2_ or CoCl_2_ with *Ctr1* siRNA treatment. Scale bars = 50 μm. (**D**) The fluorescence density of E-cadherin and vimentin per cell were measured in each groups. Data was shown as mean ± SD of three independent experiments. *p* < 0.05 was considered statistically significant (^*^compared to the control group and ^#^compared to the CoCl_2_ group).

To further confirm the roles of copper in EMT of MCF7 cells induced by CoCl_2_, *Ctr1*, a transmembrane protein responsible for cellular copper uptake, was silenced by siRNA to mimic TEPA treatment (Fig. S2). *Ctr1* silencing inhibited the elongated and spindle-like mesenchymal change, and maintained actin filaments organized in cortical thin bundles similar to that in control group (Fig. 7A). To determine the effects of *Ctr1* silencing on the expressions of the EMT makers, E-cadherin (Fig. 7B) and vimentin (Fig. 7C) were studied using immunofluorescence staining. Consistent with the effects of TEPA on EMT, *Ctr1* silencing significant abrogated CoCl_2_-induced downregulation of E-cadherin and upregulation of vimentin (Fig. 7D).

### HIF1-α-Snail/Twist1 signaling participates in the inhibition of CoCl_2_-induced EMT by TEPA

**Figure 8:**
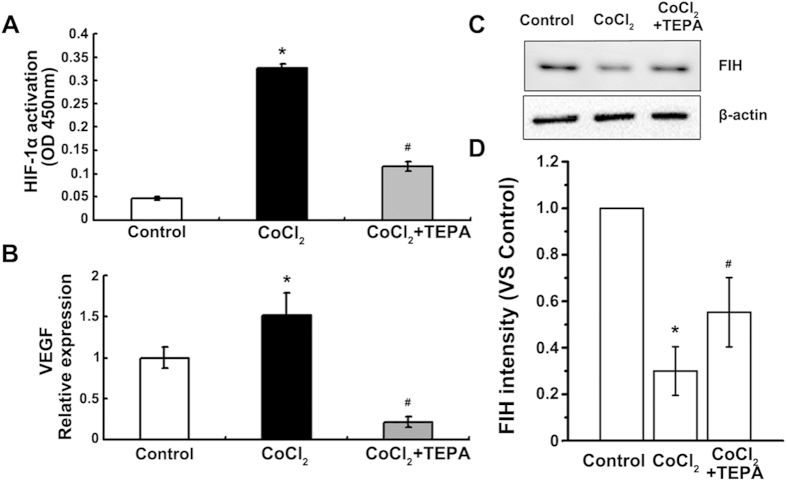
TEPA inhibits CoCl_2_ reaction of MCF-7 cells. (**A**) HIF-1 activation assay of MCF-7 cells under control, 200 μM CoCl_2_ or treatment with 50 μM TEPA. (**B**) Expression of VEGF by real time-PCR after 24 h culture. Each treatment was in triplicate and experiments were repeated at least three times. (**C**) Expression of FIH was detected by Western blotting. (**D**) The histograms showed the quantified results of the expressions of FIH, which were normalized with corresponding β-actin. Data was shown as mean ± SD of three independent experiments. In all the statistical data, *p* <0.05 was considered statistically significant (^*^compared to the control group and ^#^compared to the CoCl_2_ group).

**Figure 9:**
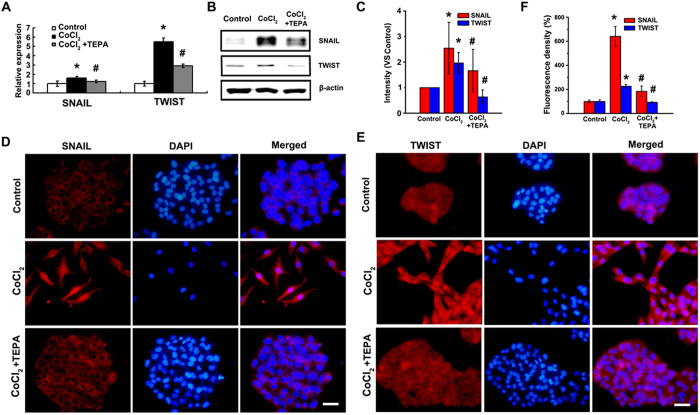
Expression and localization of Snail and Twist1 after TEPA treatment. (**A**) Expression of Snail and Twist1 by real time-PCR after 24 h culture. Each treatment was in triplicate and all experiments were repeated three times. (**B**) Protein level of Snail and Twist1 detected by Western blotting. (**C**) The histograms showed the quantified results of the expressions of the target proteins, which were normalized with corresponding β-actin. Data was shown as mean ± SD of three independent experiments. (**D**) Expression and cellular localization of Snail after 24 h incubation under control, 200 μM CoCl_2_ or treatment with 50 μM TEPA in MCF-7 cells by indirect immunofluorescence staining. Scale bars = 50 μm. (**E**) Expression and cellular localization of Twsit1 after 24-h incubation of MCF-7 cells under control, 200 μM CoCl_2_, or CoCl_2_ with TEPA, by immunofluorescence staining. Scale bars= 50 μm. (**F**) The fluorescence density of E-cadherin and vimentin were measured in each groups. Data was shown as mean ± SD of three independent experiments. In all statistical data, *p* < 0.05 was considered statistically significant (^*^compared to the control group and ^#^compared to the CoCl_2_ group).

To explore the molecular mechanisms of TEPA-mediated inhibition of CoCl_2_-induced EMT, HIF1-α-Snail/Twist1 signaling pathway was studied. TEPA treatment inhibited the activation of HIF (Fig. 8A) that was induced by CoCl_2_; significantly down-regulated the expression of VEGF, a molecule downstream of HIF1-α (Fig. 8B); and inhibited the down-regulation of FIH-1 induced by CoCl_2_ (Fig. 8C,D). Furthermore, TEPA treatment abrogated the CoCl_2_-induced up-regulation of two transcription factors, Snail and Twist1 that are critical regulators of EMT in hypoxia (Fig. 9A–C). Immunofluorescence data also showed that TEPA treatment inhibited nuclear translocation of Snail and Twist1 (Fig. 9D–F). These results indicate that TEPA inhibits CoCl_2_-induced EMT might through HIF1-α-Snail/Twist1 signaling pathway.

### Effect of copper depletion on the tumor growth *in vivo*

**Figure 10:**
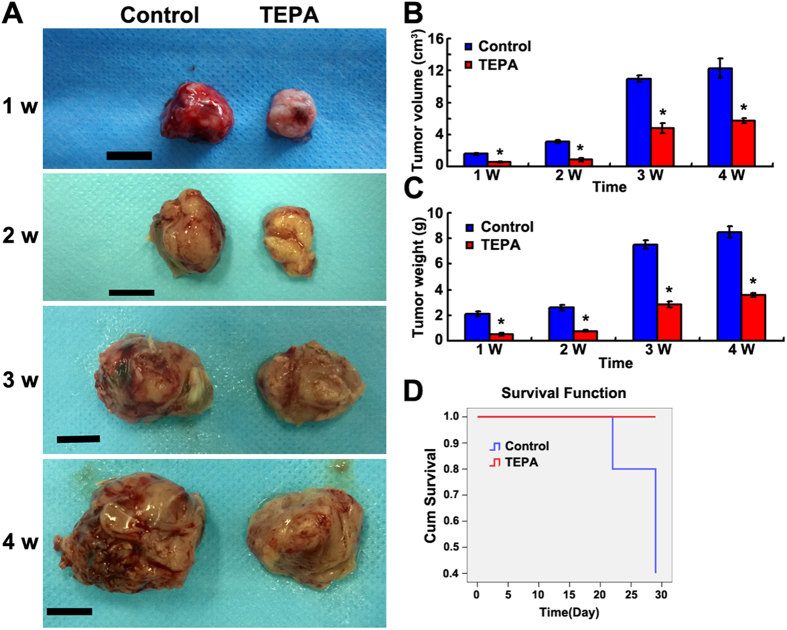
Effect of TEPA on EMT6 breast cancer in a xenograft mouse model. (**A**) Images of subcutaneous EMT6 breast tumors after oral TEPA treatment. Scale bars = 1 cm. (**B**,**C**) Evaluation of tumor growth and tumor weight at different times. ^*^p <0.05 compared to the control group. (**D**) Survival curves of mice bearing subcutaneous EMT6 breast cancer with TEPA treatment.

**Figure 11:**
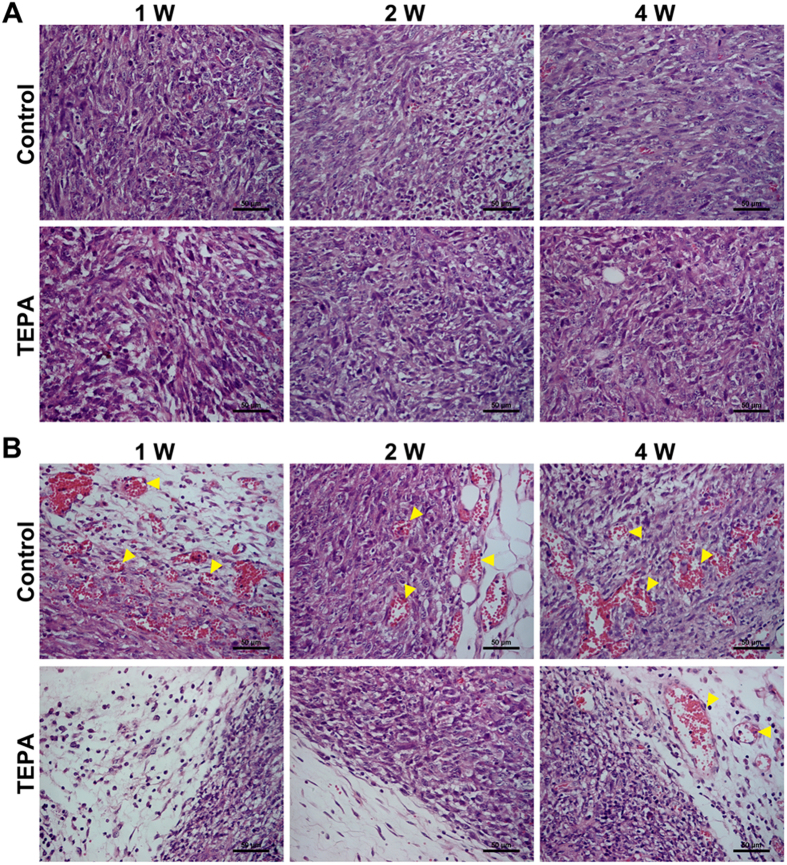
TEPA suppresses tumor angiogenesis *in vivo* in xenograft mouse model. (**A**) HE staining of tumors in control and TEPA treatment group. Scale bars = 50 μm. (**B**) Vascular formation in tumors. Shown are inhibited angiogenesis in TEPA treatment group, indicated by arrows. Scale bars = 50 μm.

**Figure 12:**
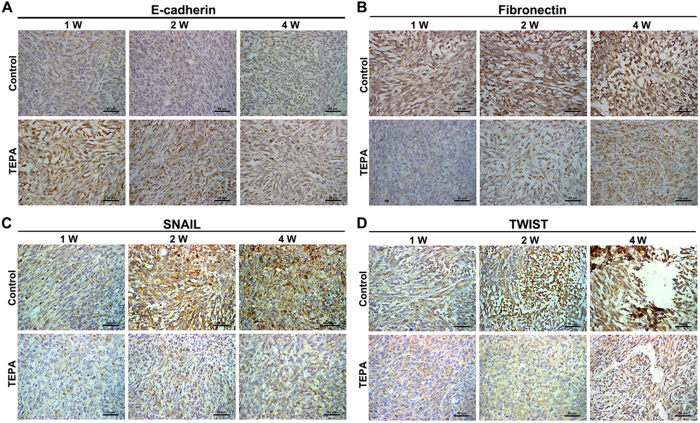
TEPA inhibits EMT marker genes expression and EMT-related transcription factors *in vivo*. Expression of E-cadherinin (**A**) and fibronectin (**B**) in tumors were determined by immunohistochemistry. E-cadherinin overexpressed after TEPA supplement while fibronectin down-expressed in tumor tissues. Expression of Snail (**C**) and Twist (**D**) in tumors were determined by immunohistochemistry. Snail and Twist positive cells were decreased by TEPA treatment. Scale bars = 50 μm.

To evaluate the effect of copper depletion on tumor growth *in vivo*, female mice were inoculated subcutaneously with EMT6 breast cancer cells and tumor growth was monitored for up to 4 weeks. Administration of TEPA significantly inhibited the growth of the EMT6 tumor compared to the control group (p < 0.01) (Fig. 10A–C), and significantly increased the survival of mice bearing EMT6 tumor (Fig. 10D). To evaluate the effect of TEPA on angiogenesis, areas with similar oxygenation, typically about 3 mm deep inside tumor from periphery, were chosen for histology and immunohistochemical studies. Although there was no difference between TEPA and control in HE staining (Fig. 11A), the number of blood vessels was significantly less in TEPA treated group compared to control (Fig. 11B). Consistent with our *in vitro* experiments, TEPA treatment increased E-cadherin expression (Fig. 12A) and decreased the expression of fibronectin (Fig. 12B), Snail (Fig. 12C), and Twist1 (Fig. 12D). These results indicate that copper-mediated activation of Snail and Twist1 play an important role in the EMT of EMT6 breast cancer cells *in vivo*.

## Discussion

In the present study, we assessed the effects and mechanisms of copper chelator, TEPA on CoCl_2_-induced EMT of human breast carcinoma MCF-7 cells. We demonstrated that TEPA down-regulated CoCl_2_-induced EMT regulators, HIF-1α, Snail, and Twist1, which in turn bind to the promoters of their downstream genes including vimentin, fibronectin, and E-cadherin, leading to the reversal of EMT phenotype and decreased migration and invasiveness of human breast carcinoma MCF-7 cells. These copper-mediated CoCl_2_-induced EMT were further confirmed by knocking down the expression of *Ctr-1,* gene coding for membrane copper transporter that is essential for cellular copper uptake. In a xenograft mouse model, TEPA inhibited the progression of EMT6 breast carcinoma and increased the survival of mice.

TEPA was extensively used to manipulate cellular copper concentrations *in vitro*^34^,^35^ and *in vivo*^36^, without affecting the cell phenotype. It is demonstrated that the affinity of TEPA for copper is significantly higher Zinc and Iron^37^. In our study, TEPA was used as copper chelator with a final concentration of 50 μmol/l, TEPA treatment only do not affect the cell morphology and EMT phenotype (Fig. S3). As a highly specific chelator of copper, TEPA could not only decrease cellular copper level but also block the activity of copper-requiring enzymes such as cytochrome c oxidase and copper-zinc superoxide dismutase^38^. And the effects of TEPA on copper levels and related enzymes activity could be reversed by copper supplementation.

Copper, as a finely regulated mineral, is essential for cellular respiration, antioxidant activity, neural synaptic function, and angiogenesis. Aberrantly increased copper has been shown to be associated with increased angiogenesis and metastasis in tumor, inflammatory diseases, and Alzheimer’s disease^2^,^39^. However, the concentration of copper for increased angiogenesis in tumor is much higher than that in physiological need, which provides a safe window for inhibiting tumor angiogenesis without affecting other normal cellular functions^16^. In deed, copper chelators have been tested increasingly in clinical trials to treat patients with advanced metastatic malignancy and showed significantly decreased tumor progression and increased survival^5^,^11^,^16^,^40^. In our xenograft mouse model, we observed significantly decreased tumor size as early as 1 week of oral TEPA administration at 1 mg/mice/day, and immunocytochemistry of the xenografted tumor showed fewer blood vessels than that in control mice. In addition to this anti-angiogenic effect of TEPA, we explored whether TEPA had any direct effect on tumor cells and the surrounding microenvironment under hypoxia.

Hypoxia, a commonly observed phenomenon in the microenvironment of solid tumor, is involved in the establishment, progression, and metastasis of tumor. Hypoxia induces hypoxia-inducible factors including HIF1-α, which exert their pathological effect through a hypoxia response element^11^ located in the promoter region of their downstream genes. HIF-1 activation has been documented and the level of its expression is directly correlated with tumor metastasis and prognosis in many tumors including both lymph-node positive and lymph-node negative breast carcinoma^41^. As a hypoxia-mimetic, CoCl_2_ has been demonstrated to stabilize HIF-1, which is associated with various hypoxic response. In most cell types, either hypoxia or CoCl_2_ could induce the main regulator HIF-1 of transcriptional responses to reduced oxygen tension. According to other reports, both hypoxia and CoCl_2_ could stimulate cancer cells EMT through HIF-1 signal pathway. Zhang and co-workers found that 1% oxygen and 100 μM CoCl_2_ treatment for 24 h could both induce the EMT of HepG2 cells^42^. Similar results were reported by other researchers^22^,^43^. Herein, in this study, we also used CoCl_2_ to investigate the effect of Cu depletion on the HIF-1 related EMT changes in human breast cancer cells, in despite of CoCl_2_ treatment cannot completely simulate hypoxic responses.

Copper acts as a cofactor for HIF-1α binding to the HRE and thus transactivates its target genes involved in EMT regulation^24^. The abundance of HIF-1α is mainly dependent on its stability and intracellular accumulation. The stability of the HIF-1α is regulated by oxygen-dependent prolyl 4-hydroxylation catalyzed by HIF prolyl hydroxylases (PHDs). It was reported that CoCl_2_-induced hypoxia can both increase protein expression of Ctr1 and copper intake in cells^44^. And this elevated copper level could lead to inhibition of PHD by Cu^2+^ salts and also contribute to the accumulation of HIF-1 protein^26^. Therefore CoCl_2_-induced hypoxia with inhibition of PHDs activity might related with copper level in cells. In our *in vitro* model, we demonstrated that hypoxia-induced by CoCl_2_ showed increased HIF1-α accumulation and HIF activation in MCF-7 breast cancer cells, which in turn promoted the EMT. In agreement, depletion of copper by treatment the cells with TEPA for 24 h significantly decreased the accumulation and activation of HIF1-α, and reversed the EMT. These results support the argument that CoCl_2_-induced EMT is mediated by aberrantly increased copper.

The signaling pathways leading to hypoxia-induced EMT remain controversial. Lester and colleagues reported that hypoxia-induced EMT of breast cancer cells was accompanied by increased expression of the urokinase-type plasminogen activator receptor and activated downstream signaling factors, including Akt and Rac1^43^. In hypoxia-induced renal tubular EMT model, biliverdin reductase was significantly upregulated and was accompanied by reduced expression of E-cadherin and increased expression of the mesenchymal marker vimentin, which could be reversed by inhibiting PI3K/Akt-dependent pathway^45^. In cancer cells of epithelial origin, hypoxia-induced EMT was mediated by transiently increased ROS at early stage and was sustained at late stage by HIF-1α-dependent expression of VEGF^23^.

Snail, a transcriptional factor, plays a critical role in the occurrence and development of cancer. It is well known that hypoxia induces the expression of HIF1-α that promotes EMT through transactivation of EMT regulators including Snail and Twist transcription factors^44^,^45^. As a critical target gene for HIF^46^, overexpression of Snail could induce EMT and is associated with highly invasive tumors both in mice and humans^47^. Increased Snail expression has been reported in hepatocellular carcinoma, glioma, and cancers of colon, cervical, and ovaries^22^. In ovarian cancer cells, hypoxia increases Snail levels and decreases E-cadherin expression^48^. Be regulated through transcriptional regulation by HIF-1α, hypoxia induced Snail expression in pancreatic cancer cells^49^. Forced expression of Snail has been associated with increased metastasis of breast cancer, whereas silencing of Snail could decreases breast cancer cell motility and invasiveness^50^. It is also reported in breast cancer cells, Snail expression in protein and mRNA level was upregulated by hypoxia treatment^33^,^43^ or CoCl_2_ supplement through elevated HIF1-α^42^. In addition to Snail, HIF-1 could also regulates the expression of Twist by binding directly to the HRE in the Twist proximal promoter and promote EMT and metastastic phenotypes^51^,^52^. In our CoCl_2_ induced hypoxia cell model, EMT was observed with concomitant increased Snail expression after 24 h. Treatment with TEPA reversed the EMT and significantly decreased the expression of Snail at both mRNA and protein levels. Twist, another transcription factor downstream of HIF1-α, was also significantly up-regulated upon hypoxia and decreased when cells treated with TEPA. Therefore, inhibition of EMT of breast cancer cell by TEPA is most likely through de-stabilization of HIF-1α and thus down-regulation the expression of Snail and Twist.

Cytoskeleton remodeling is critical during normal development, and is also a common phenomenon observed during EMT and in cancer metastasis^53^. Generally, actin reorganization in cancer cells occurs before cadherin switching and invasion^53^. In tumor cells of epithelial origin, actin filaments are organized in cortical thin bundles, whereas in trans-differentiated mesenchymal-like cells actin filaments are bundled into thick contractile stress fibers at the ventral cell surface^54^. Inhibition of PHDs by dimethyloxaloylglycine stabilizes HIF-1α, which then caused cytoskeletal remodeling in endothelial cells^55^. We demonstrated hypoxia induced irregular pattern of F-actin fibers organized in cortical thin bundles in 24 h, whereas F-actin was organized in thick stress fibers oriented in parallel which bundled at the ventral cell surface when treated with TEPA, suggesting cytoskeletal remodeling is one of the mechanisms contributing to hypoxia-induced EMT.

**Figure 13:**
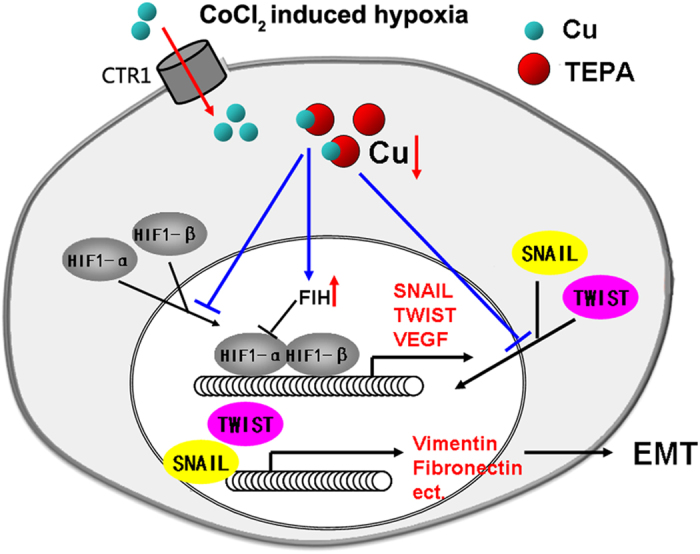
Schematic diagram of TEPA regulating CoCl_2_-induced EMT. When treated with CoCl_2_, HIF-1α subunit in cells becomes stable, and then translocates into the nucleus. HIF-1 binds to DNA and starts to regulate the targeting genes expression, and induces EMT occurrence. TEPA could chelate copper to suppress HIF1-α accumulation and activation, along with increase nuclear translocation of Snail and Twist. Consequently, EMT is inhibited by copper depletion.

Copper delivery into cells is normally mediated by a homotrimeric copper transporter, *Ctr1*^56^. When copper is uptaken into the cytoplasm, chaperones such as the copper chaperone for superoxide dismutase 1 (CCS) help to deliver copper to specific targets. Copper is required for binding of HIF-1 to hypoxia-responsive element in promoters of HIF-1 target genes, which is also CCS-dependent. As demonstrated in this study, copper chelator suppressed EMT of human breast cancer cells (Fig. 13). When the cells were cultured in hypoxia-inducing medium, higher expression of EMT regulators was observed, whereas copper depletion by TEPA led to decreased HIF-1α accumulation, increased the expression of FIH-1, and down-regulated Snail and Twist1, and consequently leading to the inhibition of EMT of breast cancer cells.

In summary, we demonstrated that TEPA inhibits hypoxia-induced EMT through targeting HIF1-α-Snail/Twist signaling pathway, which inhibits the EMT markers (vimentin, and fibronectin) and induces cytoskeleton remodeling, leading to the reversal of EMT, decreased invasiveness of malignant tumor, and enhanced survival. In light of copper chelators have been extensively used for cancer treatment for their anti-angiogenic effect, our data provided new insight into the mechanisms of copper depletion in cancer treatment. Taken together, targeting copper is a very promising treatment option for malignant tumors and copper chelators merit clinical consideration for the treatment of breast and other cancers.

## Additional Information

**How to cite this article**: Li, S. *et al.* Copper depletion inhibits CoCl_2_-induced aggressive phenotype of MCF-7 cells via downregulation of HIF-1 and inhibition of Snail/Twist-mediated epithelial-mesenchymal transition. *Sci. Rep.* **5**, 12410; doi: 10.1038/srep12410 (2015).

## Supplementary Material

Supplementary Information
