# Supplementary material for: Copper depletion inhibits CoCl2-induced aggressive phenotype of MCF-7 cells via downregulation of HIF-1 and inhibition of Snail/Twist-mediated epithelial-mesenchymal transition
Source: Sci Rep. 2015 Jul 15;5:12410. doi: 10.1038/srep12410 (PMC4502431; doi:10.1038/srep12410)
Supplement: Supplementary Information [file srep12410-s1.pdf]

Copper depletion inhibits $CoCl_2$-induced aggressive phenotype of MCF-7 cells via downregulation of HIF-1 and inhibition of Snail/Twist-mediated epithelial-mesenchymal transition

Shun Li [1], Jing Zhang [1], Hong Yang [1,2], Chunhui Wu [1,2], Xitong Dang [3], Yiyao Liu [1,2 *]

[1] Department of Biophysics, School of Life Science and Technology, [2] Center for Information in Biomedicine, University of Electronic Science and Technology of China, Chengdu 610054, Sichuan, P.R. China. [3] Division of Trauma, Surgical Critical Care and Burns, University of California San Diego, CA 92103, USA.

Table S1. Primer sequences for real time-PCR analysis

Gene	Primer sequence (5'→3')
β-actin	Forward: GGCATCCTCACCCTGAAGTA
	Reverse: GGGGTGTTGAAGGTCTCAAA
vimentin	Forward: CTCCGGGAGAAATTGCAGGA
	Reverse: TTCAAGGTCAAGACGTGCCA
VEGFa	Forward: GGCTTTTCTGTGGCAAGAGGTT
	Reverse: CGCTGATGTTTGCTCAAGTGGTC
fibronectin	Forward: GACCTATCCAAGCTCAAGTGGT
	Reverse: ATCCAAGGTTTCTGGGTGGG
Snail	Forward: TGCCCTCAAGATGCACATCCGA
	Reverse: GGGACAGGAGAAGGGCTTCTC
Twist1	Forward: GCCAGGTACATCGACTTCCTCT
	Reverse: TCCATCCTCCAGACCGAGAAGG

Sequences used for *Ctr1* silence

Primer sequence (5'→3') : GGAAGAAGGCAGUGGUAGUdTdT

Quantitative analysis of immunofluorescence data with CoCl2 or TEPA treatment.

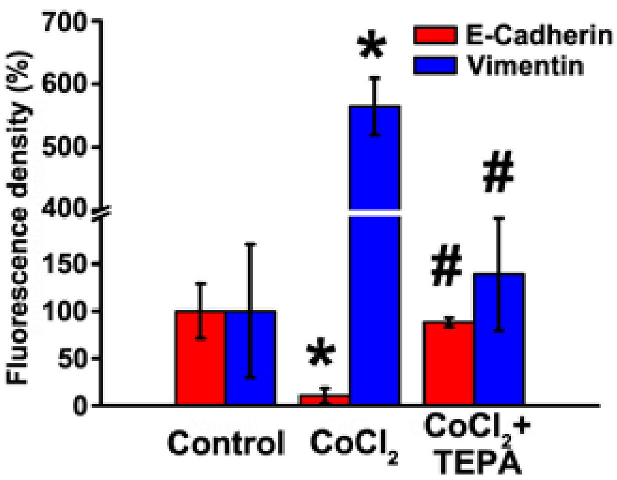

Figure S1. The fluorescence density of E-cadherin and vimentin were measured in each groups. Data was shown as mean ± SD of three independent experiments. $p<0.05$ was considered statistically significant (*compared to the control group and #compared to the $CoCl_2$ group).

Gene silence efficacy validation of *Ctr1* by western blot.

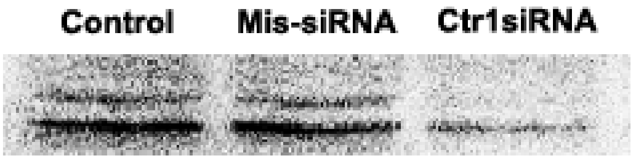

Figure S2. Gene silence efficacy of *Ctr1*. *Ctr1* expression was assayed by western blot after transfection and continued to culture for 24 h.

Morphology of MCF-7 cells under TEPA treatment only.

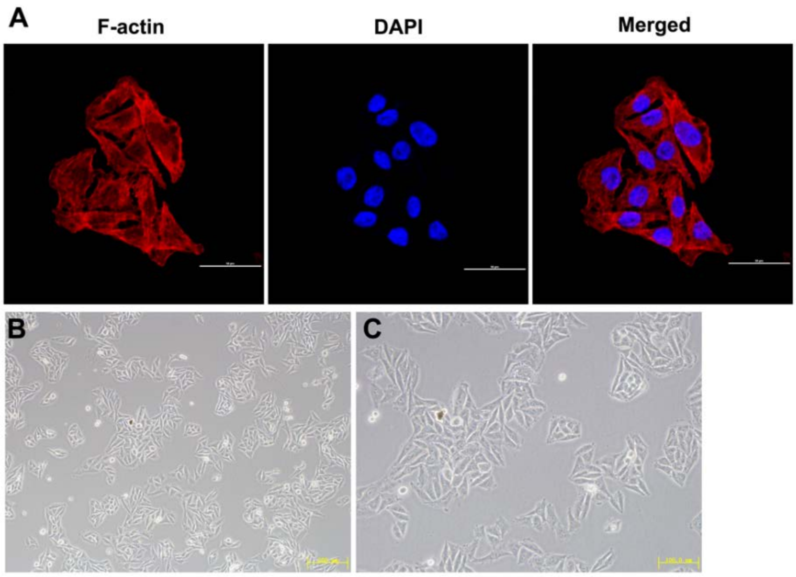

Figure S3. Morphology of MCF-7 cells under TEPA treatment only. (**A**) Spindle-shaped cells were not observed after TEPA treatment for 24 h. F-actin was stained by TRITC-phalloidin (red). Scale bar=50 μm. (**B**) and (**C**) The morphology of MCF-7 cells were visualized by phase-contrast.
